# From Decay to Delight: A Case Report on Esthetic Rehabilitation in Early Childhood Caries

**DOI:** 10.7759/cureus.67005

**Published:** 2024-08-16

**Authors:** Khushi S Zanwar, Monika Khubchandani, Manoj Chandak, Jigna Kachaliya, Ramakrishna Yeluri, Meenal S Pande, Rohan R Khetan

**Affiliations:** 1 Department of Pedodontics and Preventive Dentistry, Sharad Pawar Dental College, Datta Meghe Institute of Higher Education and Research, Wardha, IND; 2 Department of Conservative Dentistry and Endodontics, Sharad Pawar Dental College, Datta Meghe Institute of Higher Education and Research, Wardha, IND; 3 Department of Pediatric and Preventive Dentistry, Sharad Pawar Dental College, Datta Meghe Institute of Higher Education and Research, Wardha, IND

**Keywords:** esthetic, composite resin, fixed functional space maintainer, stainless steel crown, early childhood caries

## Abstract

Early childhood caries (ECC) continues to be a major global dental health concern for young children. This case report examines a young patient's transformation from decay to delight via comprehensive esthetic treatment. ECC in a four-year-old child resulted in significant dental caries and poor esthetics. In addition to space maintenance and orthodontic examination, essential treatments included dental extractions, stainless steel crowns, and composite restorations. The result showed that the child's smile and self-esteem had been successfully restored, as well as their oral health and function. This case emphasizes the value of early intervention, multidisciplinary teamwork, and individualized treatment plans in the management of early childhood caries and the restoration of pediatric patients' oral health.

## Introduction

Dental caries remains the most widespread chronic disease during childhood, presenting a significant public health concern [1]. It affects a substantial portion of school-age children, ranging from 60% to 90% [2]. According to a systematic review, the overall prevalence of early childhood caries (ECC) in India has been reported to be 49.6% [3]. It has been observed that 44% of eight- to 48-month-olds have ECC, and 40.6% of 0-3-year-old children living in rural areas of south India have been found to have the disease, with 50.3% of them having surfaces that are non-cavitated and 49.7% having cavitated surfaces [4]. ECC has a complex etiology. The condition results from an interaction among three factors, i.e., a cariogenic micro-organism (*Streptococcus mutans*), fermentable substrate, and susceptible host. The primary cause of ECC is extended bottle feeding, which includes sweetened milk, fruit juice, and pacifiers dipped in honey. Other contributing factors include a high-sugar diet and dental neglect. The condition is inherently aggressive, advancing rapidly if left untreated. Failure to halt the progression of the disease can lead to significant local, psychological, systemic, esthetic, and social consequences affecting the long-term quality of life of the child [5]. The maxillary primary incisors are primarily affected by ECC. It can result in pulpal involvement and destruction of the coronal tooth structure if the treatment is not initiated [6].

ECC develops soon after tooth eruption, grows rapidly on smooth surfaces, and causes damage to the dentition. In most cases, only the root and a small amount of the crown remain after the tooth structure is destroyed. Thus, extracting these teeth is typically the only remaining choice [7]. Adhesive restorations, like direct composite resin veneers, offer a way to restore the teeth while preserving as much natural tooth structure as possible through minimally invasive preparations [8]. In more severe cases that require extractions, it's essential to maintain the space both functionally and esthetically with an appropriate space maintainer. This rehabilitation process should ensure long-term durability without disrupting the normal eruption process of the succedaneous and adjacent teeth [9].

## Case presentation

**Figure 1:**
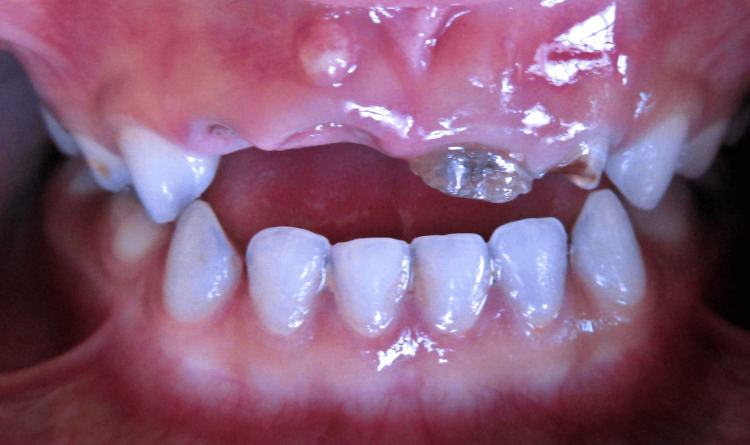
Sinus tract with 51, grossly carious 52, 61, and 62.

**Figure 2:**
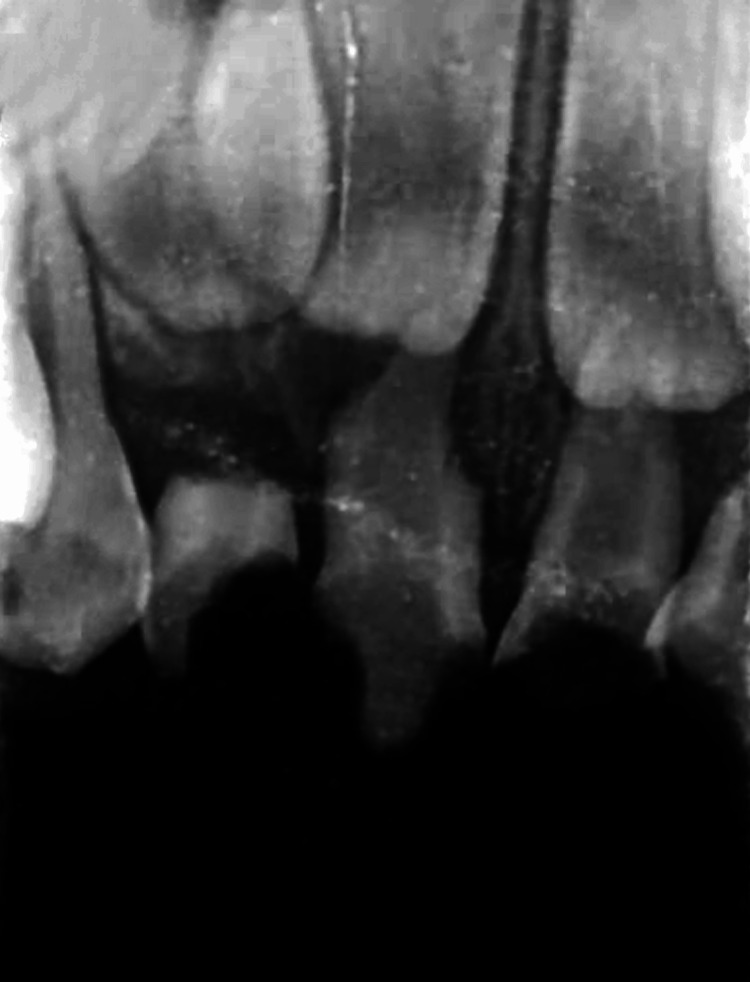
Pre-operative radiograph showing grossly decayed maxillary anterior teeth.

A four-year-old child was accompanied by his mother to the Department of Pediatrics and Preventive Dentistry with a complaint of multiple decayed teeth and an unpleasant appearance. The patient’s mother reported that the child had pain in the upper front teeth for two months. The child's mother stated during anamnesis that her pregnancy was uneventful and that she received no medical or dental care. The mother gave birth to the child normally at nine months of pregnancy. The patient had a satisfactory past medical history and overall health status. Medical and dental histories revealed no contraindications to dental treatment. Regarding nutrition, the patient's mother stated that the infant started drinking a baby bottle throughout the day after being weaned from the breast at the age of two years and eight months. Usually, sweetened milk was its contents. The frequency of bottle feeding was two to three times a day. Dietary intake was assessed using a 24-hour dietary recall. A three-day diet record indicated every other day the frequency of eating or drinking sugar-containing snacks or beverages with low nutritional content between meals, revealing six daily sucrose episodes. Oral hygiene was performed by the child using toothpaste under the supervision of the mother. During clinical examination of soft tissue, an abscess was associated with 51 (Figures 1, 2).

**Figure 3:**
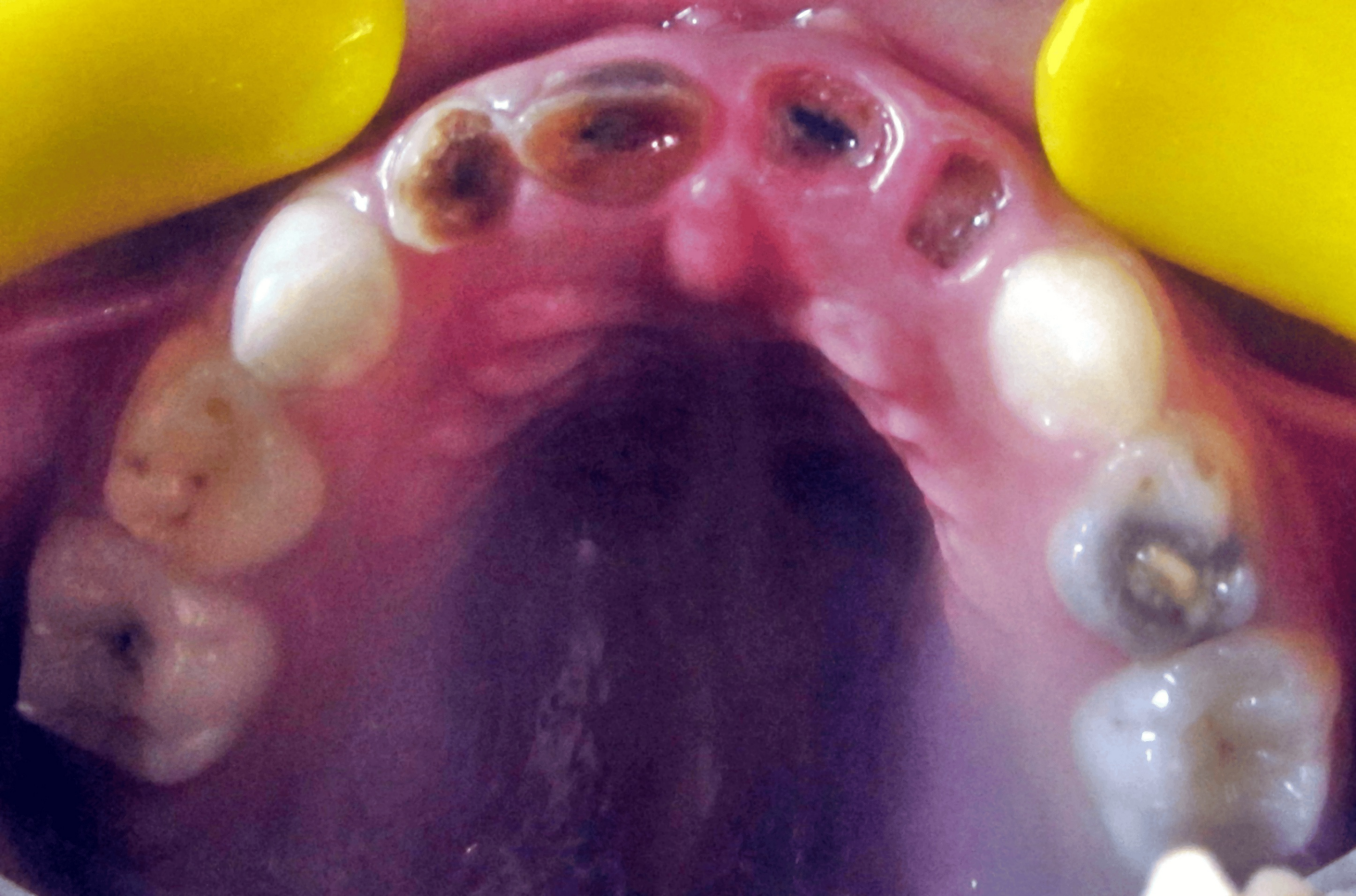
Grossly carious with 51, 52, 61, 62, deep occlusal caries with 54, pit caries with 64, 65.

**Figure 4:**
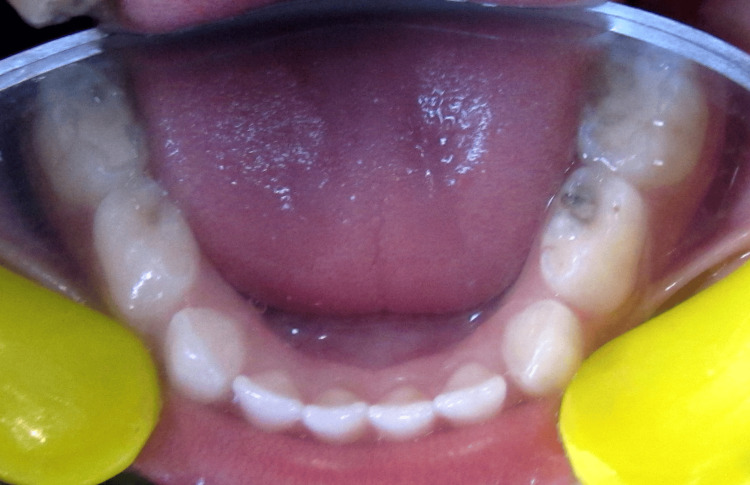
Occlusal caries with 74, tooth-colored restoration with 75 and 85 and pit caries with 84.

**Figure 5:**
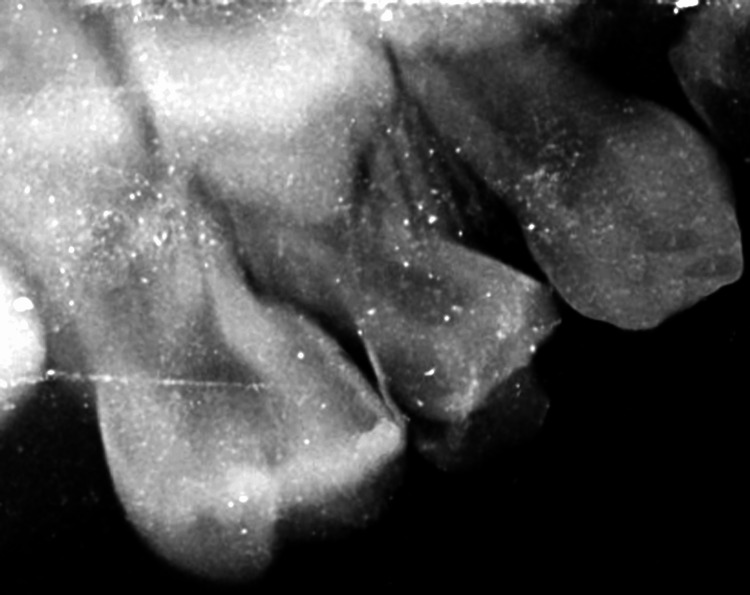
Pre-operative radiograph showing grossly carious 54.

In the examination of dental hard tissues, the following alterations were noticed; extensive coronal destruction due to caries of #51, #52, and #61. Deep caries were noted on #62 and #54, indicating pulpitis. The white opaque rough spot was seen on tooth #64, a soft cavity in the dentin of tooth #74. Tooth-colored restoration was seen with respect to #75 and #85. Enamel pit caries were noted with respect to #65 and #84, as shown in Figures 3-5.

**Figure 6:**
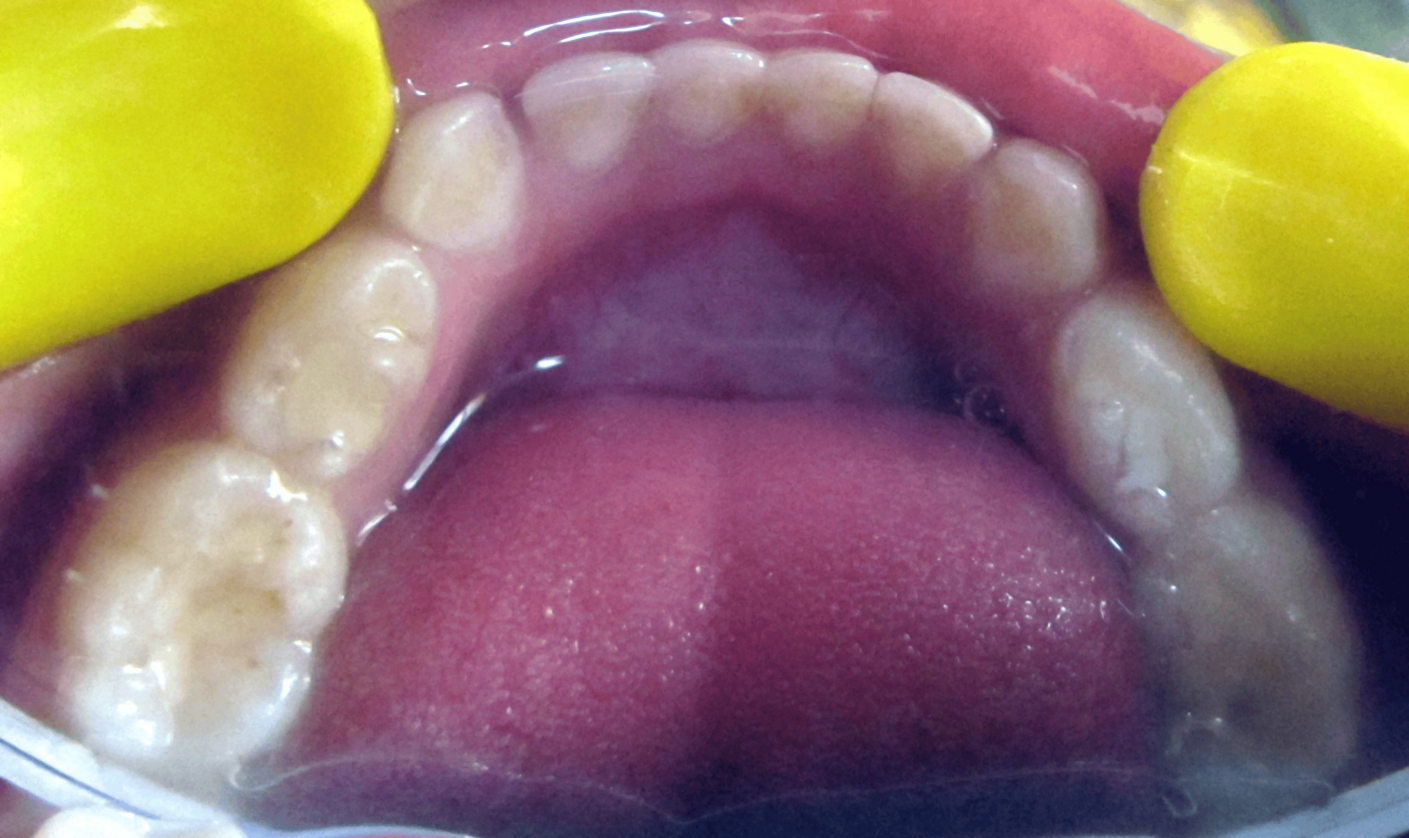
Tooth-colored restoration with 74, 75, 84, and 85.

Upon reviewing the overall examination, the final diagnosis is ECC. After completing a comprehensive case evaluation, a treatment plan was made. The extraction of grossly carious maxillary anterior teeth was followed by the placement of a functional space maintainer in order to maintain esthetics and function. The treatment plan was explained to the mother, and informed consent was obtained. The first dental treatment consisted of restorative treatment of cavitated lesions by selective caries removal for teeth #74, #84, and #65 (Figure 6).

**Figure 7:**
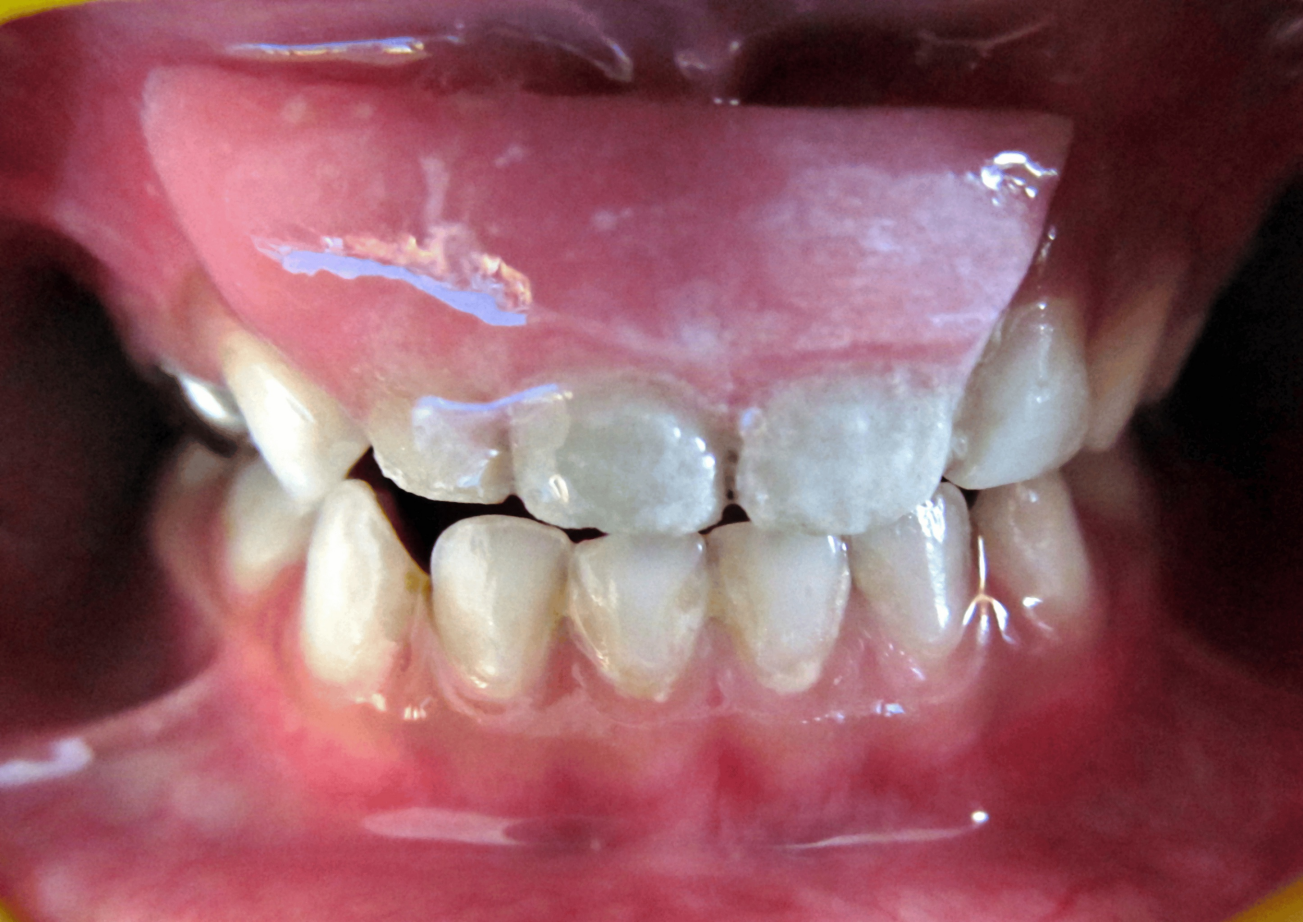
Fixed functional space maintainer can be seen with numbers 51, 52, and 61.

For teeth #51, #52, and #61, extraction followed by placement of a fixed esthetic functional space maintainer was chosen (Figure 7).

To rehabilitate #62, the treatment included pulpectomy, followed by restoration with direct composite resin veneers. It has been recommended to apply 1.23% fluoride gel topically over the course of four-week treatments in order to cure white opaque rough patch lesions on enamel. Tooth #54 was treated with pulpectomy followed by full coverage restoration with a stainless steel crown. For the maxillary anterior fixed space maintainer, a pre-operative occlusal evaluation was done. 

**Figure 8:**
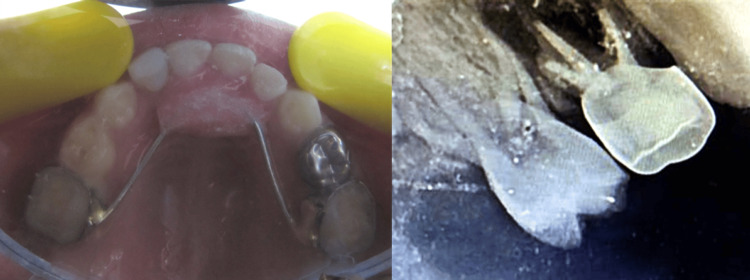
Post-operative radiograph showing pulpectomy in 54 and a stainless steel crown with 54.

Band pinching was done to teeth #55 and #65, acrylic pontics were created using self-cured acrylic, and a stainless-steel wire that extended from the anterior palatal surface was soldered to the bands simultaneously (Figure 8).

Glass ionomer cement (Vitremer, 3M Espe®, Brazil) was used to cement the prosthesis. For a month, 1.23% acidulated phosphate fluoride gel was applied once a week regularly. The family also received educational materials on diet and dental hygiene at the same time. It was recommended that the patient and their parents attend routine follow-up consultations every three months for a duration of one year in order to guarantee the best possible care and monitoring.

## Discussion

ECC is described as "the presence of one or more decayed (cavitated or non-cavitated lesions), missing teeth (cavitation), or filled tooth surfaces in any deciduous tooth in a child six years of age or younger." The presence of smooth surface caries in a child under three years of age is a marker of severe early childhood caries (sECC). The presence of one or more primary maxillary anterior teeth in an age group of 3 to 5 that are cavitated, missing (from caries), or filled, or surfaces that are decaying, missing, or filled that are ≥4 (age 3), ≥5 (age 4), or ≥6 (age 5) form sECC [10]. When tooth loss happens at least a year ahead of the regular exfoliation period, it is considered premature. Primary incisors are frequently lost between the ages of two and four years due to ECC and/or trauma. Premature loss of deciduous primary teeth could have morphological, functional, and psychological consequences. Hence, maintaining the space with a functional appliance for the development and eruption of permanent successor teeth is crucial to preventing damage [11]. 

The primary determinant of the necessity for an anterior esthetic appliance is parental desire [12]. There is no strong evidence reported in the literature to suggest that the absence of an anterior esthetic appliance will have a negative impact on the growth and development of the jaws. As per the recommendation regarding the need for maintaining space in the anterior region, space maintenance is not required if the primary maxillary and mandibular incisors are lost following the eruption of canines. On the other hand, space maintenance is required if a primary incisor is lost before the canines erupt because the primary lateral incisors may move distally and occupy the primary canine's space thereafter. In order to avoid midline deviation, the space needs to be preserved if a lower or upper primary canine is lost [13]. It has been proposed that the use of a space maintainer is not strictly necessary if only one or two central incisors are lost early. However, the provision of a space maintainer should be taken into consideration when both central and lateral incisors are lost prematurely, as it affects speech by distorting certain consonant sounds such as v, f, th, s, and z [14].

A study by Sidhom et al. reported that children with intact incisors have better production of speech than those with lost ones [15]. Another report by Kalia et al. evaluated speech before and after rehabilitation with a fixed functional space maintainer using the Weiss Comprehensive Articulation Test. There was an immediate correction in (v), (ph), (d), (dh), (th), (t), (s.), and (s) speech sounds after appliance insertion [16]. In addition to speech improvement, space maintainers also restore normal chewing function, prevent opposed teeth from supra-eruption, encourage healthy arch development, maintain esthetics, prevent the development of aberrant oral habits, and are durable and reasonably priced [8].

When a child grows from primary to mixed dentition occlusion, the dental arch undergoes modifications that may limit the use of fixed prostheses in that child. However, there exists a period of steadiness during which a fixed orthodontic appliance can be used. This stable phase typically occurs in the preschool years, during which the primary dental arch is fully developed, and the sagittal and transverse dimensions remain unchanged. In this case, the patient was four years old, falling within the indicated and stable timeframe for such an intervention [7]. Patil et al. confirmed that using a fixed space maintainer to replace a deciduous central incisor yields positive outcomes, including improved esthetics and function, reduced reliance on patient compliance, and reduced mucosal irritation [17].

Additionally, Khare et al. (2013) noted that anterior space maintainers are generally more accepted and complied with by pediatric dental patients, highlighting their wider acceptability [9]. In this particular case, a minimal amount of palatal extent was applied to minimize or eliminate mucosal irritation, and the acrylic teeth were securely supported by the gum tissue. Molars were banded instead of bonded to enhance the strength of the space maintainer. The entire process of creating and fitting the space maintainer took two appointments and was a straight-forward and quick procedure. ECC is recognized by the American Academy of Pediatric Dentistry (AAPD) as a serious chronic condition that progresses with time as a result of an imbalance of several risk and protective variables. The AAPD recommends professional and at-home preventative strategies to lower the chance of having ECC [9].

## Conclusions

In this case study, we have shown how ECC can be effectively treated using esthetic rehabilitation. We were able to successfully restore function and esthetics by combining restorative and cosmetic operations, which improved the child's smile and general well-being. This emphasizes the value of early intervention and a multidisciplinary approach to managing ECC, which will ultimately improve the quality of life and oral health outcomes for pediatric patients.
